# Checklist of the fly families Chamaemyiidae and Lauxaniidae of Finland (Insecta, Diptera)

**DOI:** 10.3897/zookeys.441.7506

**Published:** 2014-09-19

**Authors:** Jere Kahanpää

**Affiliations:** 1 Finnish Museum of Natural History, Zoology Unit, P.O. Box 17, FI-00014 University of Helsinki, Finland

**Keywords:** Checklist, Finland, Diptera, biodiversity, faunistics

## Abstract

A revised checklist of the Chamaemyiidae and Lauxaniidae (Diptera) recorded from Finland is presented.

## Introduction

Three families are currently recognized in Lauxanoidea, two of which are present in Finland. The exception is the beetle flies (Celyphidae), which are restricted to the Oriental and Afrotropical Regions.

The silvery white flies of the family Chamaemyiidae are poorly studied in Finland. Reliable identification of most species is, at least for now, only possible by the examination of male genitalia. Unfortunately many species have not been adequately (re)described which has resulted in much confusion about the proper use of species names. It is likely that several additional chamaemyiid species will be found in Finland. Of the two subfamilies of chamaemyiids, Cremifaniinae is absent from Finland. Chamaemyiinae is split into two tribes, Chamaemyiini and Leucopini, although the monophyly of the former has not yet been demonstrated (Gaimari 2010).

The lauxaniids are better known both in Finland and worldwide. Additional species are still likely to be found in the country, especially among *Homoneura* and *Sapromyza*. Of the three subfamilies, the Eurychoromyiinae, a strictly Neotropical group (Gaimari and Silva 2010a), is absent from the Palaearctic. The other two subfamilies, Homoneurinae and Lauxaniinae, are both present in Finland The subfamily Lauxaniinae is probably paraphyletic as currently defined (Gaimari and Silva 2010b).

The Finnish species of these families were last listed by Hackman (1980). Kahanpää (2013) reviewed the changes in the Finnish lauxaniid fauna since 1980.

**Number of species (Chamaemyiidae, Lauxaniidae):**

World: 336 (S. Gaimari, pers. comm. March 18, 2014), 1895 species (Pape et al. 2011)

Europe: 107, 157 species (Fauna Europaea)

Finland: 27–28, 45 species

Faunistic knowledge level in Finland: poor, average

## Checklist

suborder Brachycera Macquart, 1834

clade Eremoneura Lameere, 1906

clade Cyclorrhapha Brauer, 1863

infraorder Schizophora Becher, 1882

clade Muscaria Enderlein, 1936

parvorder Acalyptratae Macquart, 1835

superfamily Lauxanoidea Macquart, 1835

**CHAMAEMYIIDAE** Hendel, 1910

CHAMAEMYIINAE Hendel, 1910

tribe Chamaemyiini Hendel, 1910

***ACROMETOPIA*** Schiner, 1862

*Acrometopia wahlbergi* (Zetterstedt, 1846)

***CHAMAEMYIA*** Meigen, 1803

*Chamaemyia aestiva* Tanasijtshuk, 1970

*Chamaemyia aridella* (Fallén, 1823)

*Chamaemyia elegans* (Panzer, 1809)

*Chamaemyia emiliae* Tanasijtshuk, 1970

*Chamaemyia flavipalpis* (Haliday, 1838)

*Chamaemyia geniculata* (Zetterstedt, 1838)

? *Chamaemyia herbarum* (Robineau-Desvoidy, 1830) *sensu* Coe, 1943

*Chamaemyia juncorum* (Fallén, 1823)

*Chamaemyia paludosa* Collin, 1966

*Chamaemyia polystigma* (Meigen, 1830)

*Chamaemyia sylvatica* Collin, 1966

***PAROCHTHIPHILA*** Czerny, 1904

**sg. *Euestelia*** Enderlein, 1927

*Parochthiphila coronata* (Loew, 1858)

**sg. *Parochthiphila*** Czerny, 1904

*Parochthiphila spectabilis* (Loew, 1858)

tribe Lecopini Hendel, 1928

***ANCHIOLEUCOPIS*** Tanasijtshuk 1997

*Anchioleucopis geniculata* (Zetterstedt, 1855)

***LEUCOPIS*** Meigen, 1830

*Leucopis annulipes* Zetterstedt, 1848

*Leucopis argentata* Heeger, 1848

= *conciliata* McAlpine & Tanasijtshuk, 1972

= *argenticollis* auct. nec Zetterstedt, 1848

*Leucopis atritarsis* Tanasijtshuk, 1958

*Leucopis glyphinivora* Tanasijtshuk, 1958

*Leucopis griseola* (Fallén, 1823)

*Leucopis sorbi* Tanasijtshuk, 1986

Leucopis sp. cf. szepligetii Aczel, 1937

***NEOLEUCOPIS*** Malloch, 1921

*Neoleucopis atratula* (Ratzeburg, 1844)

*Neoleucopis freyi* (McAlpine, 1971)

*Neoleucopis obscura* (Haliday, 1833)

*Neoleucopis orbiseta* (McAlpine, 1971)

***LEUCOPOMYIA*** Malloch, 1921

*Leucopomyia silesiaca* (Egger, 1862)

= *alticeps* misid.

***LIPOLEUCOPIS*** de Meijere, 1928

*Lipoleucopis praecox* de Meijere, 1928

**LAUXANIIDAE** Macquart, 1835

HOMONEURINAE Stuckenberg, 1971

***HOMONEURA*** van der Wulp, 1891

*Homoneura biumbrata* (Loew, 1873)

= *tesquae* misid.

*Homoneura lamellata* (Becker, 1895)

*Homoneura mediospinosa* Merz, 2003

= *interstincta* auct. nec (Fallén, 1820)

*Homoneura tenera* (Loew, 1846)

LAUXANIINAE Macquart, 1835

***AULOGASTROMYIA*** Hendel, 1925

*Aulogastromyia anisodactyla* (Loew, 1845)

***CALLIOPUM*** Strand, 1928

*Calliopum aeneum* (Fallén, 1820)

*Calliopum elisae* (Meigen, 1826)

= *nitens* auct. nec (Loew, 1858)

***CNEMACANTHA*** Macquart, 1835

*Cnemacantha muscaria* (Fallén, 1823)

***LAUXANIA*** Latreille, 1804

**sg. *Czernushka*** Shatalkin, 2000

*Lauxania albomaculata* Strobl, 1909

**sg. *Lauxania*** Latreille, 1804

*Lauxania cylindricornis* (Fabricius, 1794)

***MEIOSIMYZA*** Hendel, 1925

= ***Lycia*** Robineau-Desvoidy, 1830 preocc.

= ***Lyciella*** Collin, 1948

*Meiosimyza affinis* (Zetterstedt, 1847)

*Meiosimyza decempunctata* (Fallén, 1820)

*Meiosimyza decipiens* (Loew, 1847)

*Meiosimyza illota* (Loew, 1847)

*Meiosimyza laeta* (Zetterstedt, 1838)

*Meiosimyza platycephala* (Loew, 1847)

*Meiosimyza rorida* (Fallén, 1820)

*Meiosimyza subfasciata* (Zetterstedt, 1838)

***MINETTIA*** Robineau-Desvoidy, 1830

**sg. *Frendelia*** Collin, 1948

*Minettia longipennis* (Fabricius, 1794)

**sg. *Minettia*** Robineau-Desvoidy, 1830

*Minettia desmometopa* (de Meijere, 1907)

*Minettia lupulina* (Fabricius, 1787)

**sg. *Plesiominettia*** Shatalkin, 2000

*Minettia helvola* (Becker, 1895)

*Minettia loewi* (Schiner, 1864)

*Minettia filia* (Becker, 1895)

**sg. unplaced in ?*Minettia*** (see Notes)

*Minettia styriaca* (Strobl, 1892)

***PACHYCERINA*** Macquart, 1835

*Pachycerina pulchra* (Loew, 1850)

*Pachycerina seticornis* (Fallén, 1820)

***PEPLOMYZA*** Haliday, 1837

*Peplomyza discoidea* (Meigen, 1830)

***POECILOLYCIA*** Shewell, 1986

*Poecilolycia vittata* (Walker, 1849)

= *quadrivittata* (Loew, 1861)

***PSEUDOLYCIELLA*** Shatalkin, 2000

*Pseudolyciella pallidiventris* (Fallén, 1820)

*Pseudolyciella stylata* (Papp, 1978)

***SAPROMYZA*** Fallén, 1810

**sg. *Nannomyza*** Frey, 1941

*Sapromyza basalis* Zetterstedt, 1847

**sg. *Sapromyza*** Fallén, 1810

*Sapromyza albiceps* (Fallén, 1820)

*Sapromyza amabilis* Frey, 1930

*Sapromyza apicalis* Loew, 1847

= *obsoleta* misid.

*Sapromyza opaca* Becker, 1895

= *imitatrix* Czerny, 1932

= *leningradensis* misid.

*Sapromyza schnabli* Papp, 1987

*Sapromyza setiventris* Zetterstedt, 1847

*Sapromyza sexpunctata* Meigen, 1826

= *atechna* Becker, 1895

= *pellucida* Becker, 1895

*Sapromyza simplicior* Hendel, 1908

*Sapromyza zetterstedti* Hendel, 1908

**sg. *Schumannimyia*** Papp, 1978

*Sapromyza hyalinata* (Meigen, 1826)

***SAPROMYZOSOMA*** Lioy, 1864

*Sapromyzosoma quadripunctata* (Linnaeus, 1767)

***TRICHOLAUXANIA*** Hendel, 1925

*Tricholauxania praeusta* (Fallén, 1820)

***TRIGONOMETOPUS*** Macquart, 1835

*Trigonometopus frontalis* (Meigen, 1830)

## Excluded species

*Homoneura dilecta* (Rondani, 1868) misidentification

*Leucopis impunctata* von Roser, 1840 nomen dubium

*Leucopis puncticornis* Meigen, 1830 nomen dubium

*Sapromyza thoracica* Becker, 1895 nomen dubium

*Neoparoecus signatipes* (Loew, 1856) not found within present borders

*Sapromyza leningradensis* Czerny, 1932 nomen dubium ?

## Notes

Several chamaemyiid species mentioned in this checklist have not been previously recorded from Finland. A paper detailing the new records is in preparation.

***Chamaemyia herbarum* (Robineau-Desvoidy, 1830)**. A poorly known species. The name has been widely used for various *Chamaemyia* species in the past. Coe (1943) and Collin (1966) applied this name (without seeing types) to a species which may be *Chamaemyia subjuncorum* Tanasijtshuk, 1980 (*sensu* Beschovski, 1995). Czerny (1936) and Tanasijtshuk (1986) listed this species as a junior synonym of *Chamaemyia juncorum* (Fallén). Most Finnish specimens under this name are either unidentifiable females or belong to other species, most frequently *Chamaemyia aestiva* Tanasijtshuk, 1970, but some specimens probably belonging to *Chamaemyia herbarum* R.-D. *sensu* Coe have been found in Finland.

**Minettia (?) styriaca (Strobl, 1892)**. This species, originally described in *Sapromyza*, has traditionally been placed in *Minettia*. Unfortunately Strobl’s description, which was based on a single female, is rather short and unillustrated. The holotype is lost according to Chvála (2008). The Finnish and Russian material in the Finnish Museum of Natural History (MZH) identified as *Minettia styriaca* (see Kahanpää 2013 for data and a photograph) matches Strobl’s description, but these specimens lack the postsutural supra-alar setae found in *Minettia* (and many other genera).
